# Where’s the “Everyday Black Woman”? An intersectional qualitative analysis of Black Women’s decision-making regarding HIV pre-exposure prophylaxis (PrEP) in Mississippi

**DOI:** 10.1186/s12889-022-13999-9

**Published:** 2022-08-23

**Authors:** Tiara C. Willie, Deja Knight, Stefan D. Baral, Philip A. Chan, Trace Kershaw, Kenneth H. Mayer, Jamila K. Stockman, Adaora A. Adimora, Mauda Monger, Leandro A. Mena, Karlye A. Philllips, Amy Nunn

**Affiliations:** 1 Department of Mental Health, Johns Hopkins Bloomberg School of Public Health, 624 N. Broadway, Baltimore, MD 21205 USA; 2 Department of International Health, Johns Hopkins Bloomberg School of Public Health, 615 N. Wolfe Street, Baltimore, MD 21205 USA; 3 Department of Epidemiology, Johns Hopkins School of Public Health, 615 N. Wolfe Street, Baltimore, MD 21205 USA; 4 Department of Medicine, Brown University, 222 Richmond St, Providence, RI 02903 USA; 5 Department of Social and Behavioral Sciences, Yale School of Public Health, 60 College Street, New Haven, CT 06510 USA; 6 Harvard Medical School and Harvard T H Chan School of Public Health, Harvard University, 25 Shattuck St, Boston, MA 02115 USA; 7 Department of Medicine, Division of Infectious Diseases and Global Public Health, University of California, San Diego, 9500 Gilman Drive, La Jolla, CA 92093 USA; 8 Department of Epidemiology, Gillings School of Global Public Health, University of North Carolina, Chapel Hill, 170 Rosenau Hall, CB#7400, 135 Dauer Drive, Chapel Hill, NC 27599-7400 USA; 9 MLM Center for Health Education and Equity Consulting Services, 123-A Hwy 80 East #258, Clinton, MS 39056 USA; 10 Department of Medicine, University of Mississippi Medical Center, 2500 N State St, Jackson, MS 39216 USA; 11 Department of Behavioral and Social Sciences, School of Public Health, Brown University, 121 S Main St, Providence, RI 02903 USA

**Keywords:** Black women, Pre-exposure prophylaxis, HIV, Racism, Sexism

## Abstract

**Background:**

Black cisgender women in the U.S. South bear a disproportionate burden of HIV compared to cisgender women in other racial and ethnic groups and in any other part of the US. Critical to decreasing new HIV infections is the improved delivery of pre-exposure prophylaxis (PrEP) for Black cisgender women as it remains underutilized in 2021. Informed by intersectionality, the study sought to characterize the sociostructural influences on Black cisgender women’s deliberations about PrEP within the context of interlocking systems of oppression including racism, sexism, and classism.

**Methods:**

Six focus groups were conducted with 37 Black women residing in Jackson, Mississippi. This sample was purposively recruited to include Black cisgender women who were eligible for PrEP but had never received a PrEP prescription.

**Results:**

Six themes were identified as concerns during PrEP deliberation among Black women: 1) limited PrEP awareness, 2) low perceived HIV risk, 3) concerns about side effects, 4) concerns about costs, 5) limited marketing, and 6) distrust in the healthcare system. Three themes were identified as facilitators during PrEP deliberations: 1) women’s empowerment and advocacy, 2) need for increased PrEP-specific education, and 3) the positive influence of PrEP-engaged women’s testimonials. Black women shared a limited awareness of PrEP exacerbated by the lack of Black women-specific marketing. Opportunities to support Black women-specific social marketing could increase awareness and knowledge regarding PrEP’s benefits and costs. Black women also shared their concerns about discrimination in healthcare and distrust, but they felt that these barriers may be addressed by patient testimonials from PrEP-engaged Black women, empowerment strategies, and directly addressing provider biases.

**Conclusions:**

An effective response to PrEP implementation among Black women in the South requires developing programs to center the needs of Black women and carry out active strategies that prioritize peer advocacy while reinforcing positive and mitigating negative influences from broader social and historical contexts.

## Introduction

Black cisgender women in the U.S. South are disproportionately affected by HIV. Cisgender women comprise one in five new HIV infections in the U.S. [1] However, Black women account for more than half (58%) of those infections [2], despite representing only 12.9% of reproductive women in the US [3]. The disparities in HIV incidence are even more significant in the Southern region of the U.S. for Black women. The Southern region of the U.S. accounts for about half of new HIV cases annually [2]. Among all women, Black women account for more than half (67%) of new HIV infections in the South and 72% of new diagnoses among women reporting heterosexual sex [1]. Moreover, the Southern region of the U.S. has the highest HIV incidence in the country with 15.6 diagnoses per 100,000 people [1]. These epidemiological data highlight the racial-, geographic- and gender-related HIV disparities facing Black women in the U.S. South, thus amplifying the need for the development and scale-up of implementation strategies of HIV prevention services specifically addressing the needs of Black women.

Prioritizing HIV prevention efforts to address cisgender heterosexual Black women is critical to address HIV incidence in the U.S. South [1]. HIV Pre-exposure prophylaxis (PrEP) is an effective HIV prevention strategy that could be used to decrease population-level HIV transmission. PrEP entails the use of a daily pill that is taken by people at risk for HIV to decrease their risk of acquiring HIV [4]. The fixed-dose combination of tenofovir disoproxil fumarate and emtricitabine is currently the only approved PrEP medication for receptive vaginal sex in the U.S. [5] To date, PrEP is underutilized among cisgender women with even fewer Black cisgender women being prescribed and initiating PrEP [6]. In 2018, approximately 5% of all PrEP users were women [6]. While the U.S. Ending the HIV Epidemic Plan aims to have at least 50% of PrEP eligible people be prescribed PrEP by 2025, the CDC estimated that only 7% of PrEP eligible women were prescribed PrEP in the U.S., with an even lower percentage for Black women, despite their disproportionate risk for HIV [7].

Specific attention to the HIV prevention needs of Black cisgender women in Mississippi is warranted. Mississippi is one of the priority states on the U.S. Plan to End the HIV Epidemic [8]. The HIV incidence rate in Mississippi is 18.6 new HIV diagnoses per 100,000 people [9], making it the state with the sixth-highest HIV incidence rate in the U.S. In addition, increasing PrEP initiation has been established as a key state priority strategy to reduce HIV transmission in Mississippi [10]. Despite these efforts, PrEP initiation remains low among Black women in the Deep South [11]. There are significant sociostructural factors that may impair access to PrEP among Black women in Mississippi such as lack of Medicaid expansion, limited health insurance coverage, stigma, and reduced healthcare capacity [12, 13]. The importance of resources to pay for PrEP is reflected by the finding that individuals who have insurance are four times more likely to use PrEP compared to those who do not have insurance [13]. In the context of geographic disparities, recent evidence suggests that the uninsured rate among Black women is highest in the Deep South (16%) [14]. There is a clear need to identify how sociostructural factors directly impact Black cisgender women’s ability to access and prioritize biomedical HIV prevention like PrEP.

### Addressing the importance of PrEP deliberation

Emerging research has delineated the unique barriers Black women face along the PrEP care continuum. For example, there is limited PrEP awareness among Black women [15, 16]. Some women also expressed disbelief that a medication to prevent HIV existed [15, 16], anger at not knowing about the medicine [17], and mistrust of the government where they assume that the medicine is being intentionally kept from them [17]. Despite initial interest, most women included in studies did not seek a prescription for PrEP and among those who did, few initiated PrEP [18]. Extant research indicates that sociostructural barriers can prevent women from initiating PrEP. Barriers to PrEP initiation included financial reasons, side effects, follow-up burden, medical mistrust, and HIV-related stigma [19, 20]. Women were also unsure of how much PrEP costs, where to go for financial assistance, if it was covered by insurance, or if they had enough money to afford the co-pay [19, 20].

The process of PrEP deliberation among Black women is a relevant but often overlooked stage when considering PrEP as an HIV prevention strategy. PrEP deliberation is a cognitive process through which constant negotiation between the benefits and the risks of taking PrEP is assessed, such as the deliberation between lower HIV risk and PrEP side effects [20]. For example, a qualitative study in New York found that women were actively engaged in an internal process of weighing the potential benefits of PrEP with the negative aspects of PrEP as well [20]. PrEP deliberation is an ongoing process and it is critical to emphasize the benefits of PrEP to Black women during this time so that they could move from PrEP deliberation to PrEP initiation. However, structural barriers must be addressed to enable Black women to do so, in particular interventions that target the earlier stages of the PrEP care continuum where the most pronounced drop-off is experienced [21].

### Intersectionality as an analytical tool to unpack sociostructural barriers of PrEP deliberation

Racial disparities in HIV incidence cannot be explained by individual differences in sexual behavior alone; research must address sociostructural barriers that increase vulnerability to HIV acquisition among Black women [22]. In particular, compared to their white counterparts, Black women are no more likely to engage in HIV-related risk behaviors, such as condomless sex with partners of unknown HIV serostatus [23]. Sociostructural factors that resulted from systemic oppression and unequal power dynamics contribute to the increased vulnerability to HIV that Black women experience. Intersectionality is a theoretical framework and can be used as an analytical tool to describe how interlocking systems of oppression and privilege interact with micro-level social identities to create multiple layers of disadvantage and inequities [24–26]. In the context of intersectionality, Black women have social identities of race and gender that reflect macro-level systems of racism and sexism. Application of an intersectional lens to PrEP inequities provides an opportunity to critically examine ways in which racism, sexism, and classism intersect to constrain Black women’s deliberation of PrEP as an HIV prevention strategy.

The processes by which sociostructural barriers influence PrEP deliberation among Black women within an intersectional context is understudied, but there are likely similarities with research examining the social context of HIV prevention [22]. Black women are socially situated at the intersection of racism and sexism. Gendered social norms, race-based stereotypes, and microaggressions can make Black women feel less empowered to make decisions about their sexual health and become more cautious when receiving new sexual health information [27]. Further, Black women are more likely to be seen as sexually promiscuous or to experience judgment from healthcare providers because of their sexual history [28]. Due in large part to racism and sexism, the history of medical experimentation and discriminatory experiences within the healthcare system has impacted Black women’s trust in medical institutions, including viewing information from providers as trustworthy [29].

Public health research that identifies how sociostructural and healthcare system factors affect Black women’s PrEP initiation could identify opportunities to improve PrEP access. In response, the purpose of this qualitative study was to characterize the experiences of Black cisgender women residing in Mississippi and assess how sociostructural and healthcare system factors influence PrEP deliberation to inform intervention strategies.

## Methods

### Participants

Between February 2019 and June 2019, six focus groups were conducted with 37 Black women in Jackson, Mississippi (*n* = 6–7 per focus group). Women were eligible for the focus group if they: (1) self-identified as a Black or African American cisgender female; (2) self-reported HIV-negative status, (3) had at least one substantial risk factor for HIV infection according to the 2017 CDC PrEP Eligibility Guidelines for heterosexual women (i.e., Sexual partner living with HIV or status unknown, diagnosed bacterial STI, 2+ sex partners, inconsistent or no condom use, sex work), (4) ≥18 years, and (5) had never taken PrEP.

### Procedures

Recruitment flyers were posted throughout the community and on social media. Interested participants calling the study line were provided details regarding the study by a research team member. If interested, they completed the eligibility screener and, if eligible, were then scheduled for a focus group. Verbal consent was obtained from the participants prior to attending the focus groups. During the focus groups, participants were asked questions regarding 1) knowledge of, attitudes towards, interest in PrEP and 2) barriers and facilitators to PrEP uptake. Notes were taken during the focus groups by a research team member. The facilitator and notetaker debriefed after the end of each focus group and developed analytical memos. Focus groups were audio-recorded and transcribed verbatim by a HIPAA-compliant transcription company and lasted on average 80 minutes. Participants were remunerated $50. The facilitators and notetakers for all focus groups were trained, Black female qualitative researchers. Focus groups were completed in person in a private room at a local community setting focused on women’s health. The Brown University and Johns Hopkins Bloomberg School of Public Health IRB approved all study procedures.

### Data analysis

To analyze the focus group data, the coding team used a general inductive approach. With a general inductive approach, data from the focus groups were coded and analyzed into themes based on the existing research objectives [30]. Initially, two focus group discussions were coded by an interdisciplinary team of three coders (i.e., public health, anthropology, and psychology). After independent parallel coding sessions, codes were discussed at team meetings to develop a codebook. This iterative process continued until a codebook was refined and finalized. After a final codebook was developed, the remaining focus group transcripts were coded by two coders. Throughout this process, the coders met regularly to discuss code application; confirm and disconfirm cases; and engage in reflexive discussion regarding assumptions and experiences. To increase the credibility of the study, community stakeholders were engaged to review data interpretation. The research team debriefed with community stakeholders regarding the discussions after each focus group session. Community stakeholders also consulted on the codebook application and theme development. Dedoose Version 4.5 [31] was used to analyze the data.

### Researcher reflexivity

The first author is a Black cisgender woman from the U.S. South who facilitated the focus groups, was one of the notetakers, and led the coding team. A co-author is a Black cisgender woman from Mississippi who facilitated focus groups, was the point of contact with the recruitment site and assisted with data interpretation. The two main coders are also Black cisgender women from the U.S. South. Information regarding the identities of the facilitators and notetakers was shared with participants during the recruitment phase.

### Findings

A total of 37 Black cisgender women participated in the focus groups. The average age of the participants was 32 years. More than half of the sample (53.8%) had an annual household income of $30,000 or less. The majority of the women were employed (80.8%), 80.8% identified as heterosexual, and 90% had received healthcare services in the past 12 months.

Across the six focus groups, Black women engaged in informative discussions regarding sociostructural and healthcare system factors shaping their deliberation and consideration of PrEP as an HIV prevention strategy. Findings from this study were informed by intersectionality [24–26] to draw attention to the sociostructural power relations that can constrain Black women’s choice to use HIV prevention.

### Concerns during PrEP deliberation

#### Limited PrEP awareness and knowledge

Consistently throughout the focus group discussions, women talked about their limited knowledge and awareness of PrEP. For example, when asked why few Black women were using PrEP, the majority of participants noted limited PrEP awareness:

**Table:** 

Participant 1:	“They just don’t know about the pill.”
Several Participants in unison,:	“Yes.” [Focus group 6]

Some women also explained that they recognized PrEP from media commercials but were still unclear about who was eligible for PrEP:

**Table:** 

Participant 1:	“I don’t know much about it [PrEP].”
Participant 2:	“Well, I’ve heard of it [PrEP]. I’ve seen commercials, TV commercials, but like I didn’t know if it was targeted towards certain people. Like how do I know that is a preventative? Or is it free for all? Because I want it.” [Focus group 1]

Several women also expressed that very few members within their social network such as family and friends, were talking about PrEP or using PrEP. In particular, when asked if they heard of anyone being on PrEP, women stated:

**Table:** 

Several Participants in unison:	“No.” [Focus group 3]

Some participants commented that they felt comfortable discussing PrEP with their healthcare providers, however, they felt that patient-provider conversations should be centered around gaining more information about PrEP:

**Table:** 

Participant 1:	“I feel okay about talking to him about it so I can get a clear understanding about it too. Like, how long do you have to take it? What’s the percentage of it actually working to prevent you from having HIV. The cost of it.” [Focus group 2]

#### Low perceived HIV risk and PrEP candidacy

Women’s perceptions of their risk for HIV acquisition were an important factor during PrEP deliberation. In particular, some women perceived their risk for HIV infection to be low due to their marital status, and thus were disinterested in PrEP and did not perceive themselves to be appropriate candidates for it. For example:

**Table:** 

Participant 1:	“Personally, I think I don’t need it. I mean I don’t consider myself being high risk.”
Participant 2:	“My partner doesn’t have it, so I don’t think I need it.”
Participant 3:	“Yeah.”
Participant 4:	“I’m the same way too.”
Participant 2:	“I don’t think I need it.”
Participant 2:	“Like we’ve said, they’re married, and I only have one partner. And we get tested once a year. And so, neither one of us is positive.” [Focus group 4]

Black women also discussed that their HIV risk perception fluctuated with their age and marital status. For example, there was discussion around PrEP being an optimal prevention strategy when women were younger and single:

**Table:** 

Participant 1:	“I mean I probably was at risk more, but didn’t know because being younger, I wasn’t—you know, I really didn’t know. I mean I heard about HIV, but, you know, it was always like that couldn’t happen to me.”
Participant 2:	“Yeah.”
Participant 1:	“So probably was at risk when I was younger, but never thought I was. As I got older now and I’m older, I mean I don’t think I’m at risk ‘cause, of course, like I said, I’m married, but probably was at risk when I was in my younger teenage days, early 20s.” [Focus group 4]

Women also shared that some Black women were not ready to admit their need for PrEP. Specifically, women may minimize their risk for HIV and thus avoid discussing or considering PrEP:

**Table:** 

Multiple Participants in unison:	“Denial.”
Participant 1:	“Yeah. No one believe it. Don’t wanna believe it.”
Participant 2:	“Yeah.”
Participant 1:	“You don’t wanna admit to yourself.”
Participant 2:	“Yeah.”
Participant 1:	“Yeah. You don’t wanna see yourself taking this medication so you won’t get something because you know that you can get it.”
Multiple Participants in unison,:	“Yes.” [Focus group 6]

#### Side effects

The impact of the potential side effects on women’s current health conditions was a barrier to PrEP initiation among Black cisgender women. It was clear that Black cisgender women needed more information regarding the side effects of PrEP in order to adequately consider the risks and benefits of using PrEP for HIV prevention. In the focus group discussions, participants noted that the limited education about the potential side effects of PrEP was an important contributor to the low rates of PrEP utilization:

**Table:** 

Participant 1:	“Now I’m kinda taking into consideration what it is I’m putting into my body and seeing what the side effects are.”
Participant 1:	“Mm-hmm, mm-hmm.”
Participant 2:	“It’s like she said, that’s why a lotta people don’t wanna take the drug.”
Several Participants in unison,:	“Yeah.” [Focus group 5]

Even among a group of participants who were knowledgeable of the potential PrEP side effects, there was some concerns about using PrEP. In particular, this group of participants expressed their concerns about how the potential PrEP side effects may negatively interact with their ongoing health conditions:

**Table:** 

Participant 1:	“I mean, all them side effects. I don’t know, they was just scaring me. I don’t know. It’s a whole list. I don’t know. Yeah, all that. My bones already poppin’ out on me.” [Focus group 3]

#### Costs related to PrEP

Focus group participants also discussed how the cost of PrEP and its associated fees (e.g., laboratory costs) may negatively impact women’s access to PrEP. In particular, women stated the cost of PrEP as an important barrier to PrEP initiation: “The cost” (Participant 1) and several particpants agreed in unison from Focus Group 6.

One participant also shared that the cost of PrEP was not a significant barrier to access PrEP, but most participants wanted to know about the different mechanisms to pay the fees related to PrEP initiation and persistence:

**Table:** 

Participant 1:	“Yes.”
Participant 2:	“Cost ain’t a reason.”
Participant 1:	“Oh, yes, it is. Hold up.”
[Laughter from Several Participants]	
Participant 1:	“Hold up. I do wanna know how much this thing costs.”
Several Participants in unison:	“Yeah.”
Participant 2:	“They [accept] Medicaid?”
Participant 3:	“Yeah.”
Participant 2:	“See we got [Insurance company]. You gonna pay for half of that, baby. They’re [Insurance company] is a little stingy.” [Focus group 3]

The challenges associated with PrEP affordability and cost were complicated by the broader sociostructural factors such as racialized gender pay inequity that participants face. This sociostructural context can constrain Black women’s ability to afford PrEP.

#### Limited PrEP Marketing for Black Women

The erasure of Black women from PrEP marketing creates more difficulties for this priority population to access biomedical HIV prevention. In general, participants discussed the lack of PrEP-related health communication strategies that are inclusive of Black women. In some focus group discussions, Black women stated they did not see themselves and their community in PrEP commercials and advertisements. So when asked about seeing themselves in commericals, participants tended to respond as:

**Table:** 

Several Participants in unison:	“No.” [Focus group 1]

Altogether, gendered racism fuels the invisibility of Black women from PrEP marketing as a sociostructural barrier with the potential to delay PrEP initiation. In multiple focus groups, participants described their perceptions of the marketing strategies for PrEP advertisements and how this limited marketing could fail to persuade Black women that they were PrEP candidates.

**Table:** 

Participant 1:	“But then like, you know, we talked about the ads that are out for PrEP. And if I’m not mistaken, one of the people is transgender. And so, I think if there was an actual Black [cis-gender] female in advertisements, and it wasn’t just gay men or transgender people who are in the ads. So, then it would make everybody feel like, okay, so everybody’s, um, you know, able to use it. It’s not just for specific people.”
Participant 2:	“All genders.” [Focus group 4]

In addition to noting the lack of diverse PrEP advertisements, participants offered potential solutions and recommendations to improve these advertisements by focusing on the “Everyday Black Woman.”

**Table:** 

Participant 1:	“There’s no commercial with a regular woman who’s dropping off kids, and who randomly meets somebody in a grocery store. I mean that ain’t what you’re seeing on the commercials.”

#### Distrust in healthcare system

Black women had PrEP concerns that also stemmed from misinformation of PrEP efficacy and its overall purpose. In particular, there was discussion on whether or not women believed PrEP could prevent HIV:

**Table:** 

Participant 1:	“If it’s really [effective]. Is it really gonna help? I’m sayin’ they have a lot of stuff out here that say that it’s gonna help you or help you, say it is gonna prevent a lot of things, but is it always 100% accurate or not?”
Participant 2:	“So, a lot of people probably just don’t wanna do it because they probably don’t think that it’s really the cure for HIV or to prevent HIV.”
Several Participants in unison:	“Yeah.” [Focus group 2]

Equally important, some women discussed the socio-historical underpinnings of medical research and practices with Black Americans. For example, these prior socio-historical practices may cause additional concerns and red flags for Black women considering PrEP:

**Table:** 

Participant 1:	“Just culturally, when you think about studies and medication, the African American community. The thinking for African Americans, they’re like, “Well, they’re probably tryin’ to kill us all. They’re tryin’ to harm us all. Why are y’all takin’ that medication?”
Several Participants in unison:	“Yeah.” [Focus group 1]

Likewise, women described their experiences of racial- and gender-related discrimination with healthcare providers as a significant barrier to preventative healthcare, including PrEP access.

**Table:** 

Participant 1:	“A lot of these health care providers are very critical and judgmental, and they don’t treat all patients fairly, you know.”
Participant 2:	“Yeah.”
Participant 1:	“They judge automatically on appearance, look, race, and sexuality.”
Participant 2:	“Yeah.”
Participant 1:	“And often, at times, they don’t get the best health care because of the health care provider.”
Participant 2:	“Yeah.”
Participant 5:	“That’s true.” [Focus group 1]

### Facilitators during PrEP deliberation

#### Increased PrEP education

Facilitators during the PrEP deliberation process that are rooted in Afrocentric principles may repel some of the negative impacts of sociostructural barriers of PrEP intiation. For instance, Black women also shared that increased education and resources on PrEP was another strategy to facilitate PrEP initiation. Increasing PrEP education and resources aligns with the Afrocentric concept of collective responsibility as this facilitator can build the community and help solve the issue of low awareness in the community.

**Table:** 

Participant 1:	“Like I said, education, groups. We need to get it out there.” [Focus group 4]

#### Women’s testimonials

Similar to increasing PrEP education, participants also described the importance of patient testimonials as a facilitator of PrEP initiation. Advocating for patient testimonials among PrEP-engaged Black women also aligns with the Afrocentric concept of collective responsibility. In particular, Black women sharing their experience of PrEP initiation with women from their community demonstrates that the larger community is taking care of each other and sharing both positive and negative experiences. For example:

**Table:** 

Participant 1:	“It’s good. I think people should try it.”
Participant 2:	“Uh, somebody gotta try it before me.”
Participant 1:	“You get my number, and I’ll try it before you. How about that?”
Participant 2:	“Okay, yeah. Yeah, if she try it, then I’m gonna catch up with her, and she just let me know how it worked for her.” [Focus group 3]

Even more specifically, women shared that patient testimonials of PrEP initiation and adherence without any side effects would be a key determinant.

**Table:** 

Participant 1:	“Yeah. If she good. Her body good and all this happened, so then, you know, I’ll just go ahead and pop my pill.” [Focus group 3]

#### Women’s empowerment and advocacy

Some Black women shared being empowered to communicate and advocate for their healthcare needs to their medical providers. Being empowered to communicate for one’s needs aligns with the Afrocentric concept of self-determination which postulates that Black women can name, define, and govern their own interests. Empowering Black women to make informed, autonomous decisions within their healthcare may help increase PrEP initiation:

**Table:** 

Participant 1:	“When I go to the doctor, I don’t care what doctor it is, they work for me. And I come in with a mindset, you work for me. I have a list of whatever I want to talk to them about. You’re not gonna come in here and see me for 30 seconds or less than a minute. You got to stay. So, usually, like if I go to the doctor, if there’s any questions I have, I would write it down and put it in my phone.” [Focus group 1]

## Discussion

This was among the first studies to evaluate PrEP deliberation among Black women in the Deep South. Guided by intersectionality [24–26], the present study assessed sociostructural influences on PrEP deliberation and initiation among Black women. Consistent with prior research [17, 32, 33], there are significant implementation barriers that impede access to PrEP among Black women. However, the present study used an intersectionality framework as an analytical tool to situate Black cisgender women’s process of PrEP deliberation within the context of interlocking systems of oppression including racism, sexism, and classism. For example, our findings indicate that women’s concerns such as limited awareness; low perceived risk; side effects, costs; limited marketing; and medical distrust, and facilitators including empowerment and advocacy; PrEP education; and testimonials are important drivers as Black women deliberate about PrEP as an HIV prevention strategy. Within an intersectional framework, narratives from Black women illustrate that interlocking systems of power and oppression (i.e., racism, sexism, and classism) can foster obstacles to PrEP initiation. Yet, solutions that galvanize an Afrocentric perspective may meaningfully address cultural values during the PrEP deliberation process to facilitate PrEP initiation. Altogether interventions aiming to enhance positive attitudes towards PrEP among Black women might be helpful, but upstream approaches such as structural and community-level interventions are needed to increase PrEP awareness, access, initiation, and retention.

In recent years, HIV prevention researchers have called for research on structural contexts [34–37], and Black women in our study described three key structural barriers to PrEP consideration and initiation: costs; limited specific marketing; and medical distrust. Financial costs have been documented as a deterrent [33, 38], but structural mechanisms such as racial- and gender-related wage gaps [39] and gendered racism [40] have contributed to economic inequalities experienced by Black women. For Black women in Mississippi, these inequalities are exacerbated by a regressive policy climate in which there is no equal pay law [41] and no Medicaid expansion [12]. Public advocacy for Medicaid expansion and universal offering of patient medication assistance programs are potential avenues to address this financial concern. Upstream approaches such as anti-discriminatory policies, equal pay laws, and increasing the minimum wage may help close inequality gaps, bolster steady revenue streams, and create sustainable access to PrEP for Black women.

At a recent CDC town hall meeting, the lack of women-specific PrEP marketing was noted as a barrier to PrEP initiation [42]; however, our focus group discussions with Black women underscore the importance of PrEP marketing that captures the lived realities of Black women. The general lack of PrEP marketing specific to Black women’s lives emphasizes how racism, sexism, and classism influence the overall de-prioritization of HIV prevention for Black women. Conversely, PrEP marketing that is tailored to Black women in clinics could enhance PrEP initiation by reinforcing that Black women are appropriate PrEP candidates; normalizing PrEP use among women; showing that healthcare providers are open to discussing this HIV prevention option with Black women. Utilizing culturally congruent PrEP marketing may help support Black women during their deliberation process as they are also navigating multiple structural obstacles.

The focus group results further illustrated the importance of medical mistrust and negative healthcare experiences as Black cisgender women deliberated about PrEP. In particular, there was general skepticism regarding PrEP’s effectiveness that could be partially assuaged with culturally appropriate health communication strategies. However, Black women discussed their negative experiences with healthcare providers stemming from provider bias and judgmental behaviors during clinical encounters. Their general skepticism in conjunction with experiences of provider bias contributes to medical mistrust and distrust in the healthcare system. Consistent with prior research on PrEP initiation among Black women [32, 33], limited PrEP awareness and low perceived risk perception for HIV acquisition appeared to delay PrEP initiation. Specifically, Black women who believe they are at low risk for acquiring HIV may view PrEP as unnecessary and thus view themselves as inappropriate candidates for PrEP. Some women in the study shared that the type and frequency of PrEP side effects could influence future PrEP initiation. Black women communicated the importance of knowing the types of side effects that could occur, but also indicated that understanding how PrEP interacted with other medications and health conditions was equally important.

Creating opportunities to promote empowerment and advocacy among Black women might facilitate PrEP initiation by addressing specific individual-level barriers. In the discussions, some participants felt empowered to advocate for their needs. Extant research demonstrates that Black women are often expected to practice self-silencing practices such that they minimize their personal needs [43]. Therefore, providing safe spaces for Black women to deliberate and make informed, autonomous decisions may enhance PrEP initiation. Healthcare providers should be trained to recognize this sociocultural challenge in Black women’s lives and offer non-judgmental, open conversations regarding sexual health. Also, culturally-congruent peer advocates and advocacy groups could be an important resource for Black women who are deliberating about PrEP and need to discuss healthcare concerns. In particular, advocates and groups can be trained to recognize and uplift Afrocentric principles (e.g., collective responsibility, self-determination) [44] as these concepts relate to the experiences of and recommendations set forth by Black women. Utilizing Afrocentric principles is important as this work centers the lived experiences of people of African descent which recognizes the collective experience of disenfranchisement [45, 46]. Emerging research has illustrated promising results with peer advocates in the context of addressing racial and ethnic health disparities [47]. Investing in Black women’s empowerment and advocacy could increase PrEP awareness and potentially promote initiation.

Despite these important findings, there are study limitations. Our sample was purposively recruited from Jackson, Mississippi, an HIV hotspot. There may be similarities in Black cisgender women’s experiences in other HIV hotspots in the Deep South. This study used the CDC guidelines to determine eligibility for PrEP, one of the study’s inclusion criteria. Recent research suggests that the CDC guidelines for PrEP eligibility may disqualify women who are motivated to use PrEP [48, 49], thus it is possible that this group of women were not representative in these focus groups. While the goal of this study was not generalizability, we strengthened the transferability of our findings by providing a rich description of not just the behaviors and experiences but also the context of Black women’s lives. Within the focus groups, differences may exist between women who were more vocal during the group discussion compared to those who were not. Facilitators were trained to recognize these situations and respond in ways that encouraged more active participation for all focus group members.

## Conclusions

There is an emerging body of research examining Black women’s interest in biomedical HIV prevention, but our study sought to understand PrEP decision-making among Black cisgender women in the U.S. South. Chiefly, structural interventions are needed to address the sociostructural barriers that Black women in the Deep South face when prioritizing and accessing PrEP. Sustainable partnerships among researchers, activists, public health agencies, and community organizations are needed to collectively invest in structural interventions that enhance access to PrEP. In 2021, significant inequities in PrEP initiation among Black women in the U.S. South continue to persist and will do so until academic, community and governmental entities begin contributing meaningfully to addressing obstacles and promoting facilitators to tackle unmet HIV prevention needs.

## Data Availability

The qualitative data generated and/or analysed during the study are not publicly available because they contain information that could compromise participant privacy and/or consent. The corresponding author can be contacted for follow-up questions and/or concerns.

## Abbreviations

PrEP: Pre-exposure prophylaxis
HIV: Human Immuno-Deficiency Virus
U.S.: United States
IRB: Institutional Review Board
HIPAA: Health Insurance Portability and Accountability Act
